# Prospective Comparative Study of EMSella Therapy and Surgical Anterior Colporrhaphy for Urinary Incontinence: Outcomes and Efficacy

**DOI:** 10.3390/healthcare13080864

**Published:** 2025-04-10

**Authors:** Geanina Sacarin, Ahmed Abu-Awwad, Nitu Razvan, Marius Craina, Mihaela Prodan, Madalina-Otilia Timircan, Razvan Betea, Anca Dinu, Simona-Alina Abu-Awwad

**Affiliations:** 1 Doctoral School, “Victor Babes” University of Medicine and Pharmacy, 300041 Timisoara, Romania; geanina.sacarin@umft.ro (G.S.); mihaela.prodan@umft.ro (M.P.); razvan.betea@umft.ro (R.B.); 2 Department XV—Discipline of Orthopedics—Traumatology, “Victor Babes” University of Medicine and Pharmacy, 300041 Timisoara, Romania; ahm.abuawwad@umft.ro; 3 Research Center University Professor Doctor Teodor Șora, “Victor Babes” University of Medicine and Pharmacy, 300041 Timisoara, Romania; 4 Clinic of Obstetrics and Gynecology, “Pius Brinzeu” County Clinical Emergency Hospital, 300723 Timisoara, Romania; craina.marius@umft.ro (M.C.); madalina-otilia.timircan@umft.ro (M.-O.T.); alina.abuawwad@umft.ro (S.-A.A.-A.); 5 Department of Obstetrics and Gynecology, Faculty of Medicine, “Victor Babes” University of Medicine and Pharmacy, 300041 Timisoara, Romania; 6 Department of Plastic Surgery, “Pius Brinzeu” Timis County Emergency Clinical Hospital, 300723 Timisoara, Romania; 7 Department of Obstetrics and Gynecology, Municipal Hospital Deva, 330084 Deva, Romania; 8 Department XVI—Medical Recovery, “Victor Babes” University of Medicine and Pharmacy, 300041 Timisoara, Romania; dinu.anca@umft.ro; 9 Research Center for Assessment of Human Motion and Functionality and Disability, “Victor Babes” University of Medicine and Pharmacy, Eftimie Murgu Square, No. 2, 300041 Timisoara, Romania

**Keywords:** urinary incontinence, cystocele, electromagnetic field therapy, pelvic floor dysfunction, pelvic organ prolapse, non-invasive therapy, vaginal surgical procedures

## Abstract

**Background:** This prospective comparative study investigates urinary incontinence (UI), often associated with grade 2 cystocele, a condition that poses significant physical, emotional, and social challenges for affected women. While anterior colporrhaphy remains the gold standard for anatomical correction, non-invasive alternatives such as EMSella therapy have gained increasing attention. The study compares the outcomes of these two distinct approaches in managing UI and the associated pelvic organ prolapse. **Materials and Methods:** This study involved 133 menopausal women with grade 2 cystocele and UI, including 78 treated with anterior colporrhaphy and 55 with EMSella therapy, across two Romanian healthcare centers. Outcomes were assessed through prolapse reduction (POP-Q), bladder function normalization, recurrence rates, quality of life (PFDI-20, PFIQ-7), patient satisfaction, complication rates, recovery times, and social or professional disruptions. **Results:** Anterior colporrhaphy was more effective in anatomical correction (88% vs. 64% achieving stage 0 prolapse) and bladder function normalization (72% vs. 55%, *p* = 0.04), with lower one-year recurrence rates (14% vs. 31%, *p* = 0.03). EMSella therapy allowed faster recovery, with 91% resuming daily activities within a week. Both groups showed improvement in quality of life, but reductions in PFDI-20 and PFIQ-7 scores were more significant after surgery. EMSella had fewer infections and no dyspareunia, reflecting a better safety profile. **Conclusions:** EMSella therapy and anterior colporrhaphy significantly benefit managing UI associated with grade 2 cystocele. While anterior colporrhaphy provides definitive anatomical correction and superior long-term outcomes, EMSella therapy represents a safer, less invasive alternative with rapid recovery, making it ideal for patients with mild conditions or surgical contraindications. Treatment should be tailored to individual patient needs and preferences.

## 1. Introduction

Urinary incontinence (UI) is a common condition affecting millions of women worldwide, with notable physical, emotional, and social consequences [1]. Defined by involuntary urine leakage, UI frequently coexists with pelvic organ prolapse (POP), particularly cystocele, where weakened pelvic floor support causes the bladder to descend into the vaginal canal [2]. Grade 2 cystocele, a moderate form, involves bladder protrusion into the vagina without extending beyond the vaginal opening, often resulting in urinary urgency, stress incontinence, and discomfort, which significantly reduce quality of life (QoL) [3]. Managing grade 2 cystocele with UI is essential for restoring bladder function, alleviating symptoms, and improving QoL.

Urinary incontinence associated with cystocele primarily manifests as stress urinary incontinence (SUI) and urge urinary incontinence (UUI). SUI results from weakened urethral support, leading to involuntary leakage during activities that increase intra-abdominal pressure, such as coughing or exercise [4]. This occurs due to loss of normal urethral positioning and sphincteric function, which are essential for maintaining continence [5]. UUI, often linked to detrusor overactivity, is characterized by sudden urgency and involuntary leakage [6]. The pathophysiology of detrusor overactivity in UUI is complex, involving both neuromuscular dysfunction and bladder hypersensitivity [7]. Additionally, in severe cases, bladder outlet obstruction may cause overflow incontinence due to incomplete emptying and subsequent urine retention [8]. Given the multifactorial nature of these symptoms, treatment must address both anatomical and functional aspects of bladder dysfunction [9].

Traditionally, surgical correction through anterior colporrhaphy has been the primary treatment for grade 2 cystocele with UI. This procedure reinforces the anterior vaginal wall, restoring bladder support and improving urethral positioning. Studies have shown that anterior colporrhaphy effectively reduces symptoms in 70–90% of cases, improving stress urinary incontinence and urgency by enhancing urethral closure pressures. However, while long-term success rates are generally favorable, recurrence occurs in up to 30% of cases, and complications such as dyspareunia, voiding dysfunction, and postoperative infections remain concerns [10,11,12].

Given the risks and recovery time associated with surgery, non-invasive treatments such as EMSella therapy have gained interest. EMSella utilizes high-intensity focused electromagnetic (HIFEM) stimulation to induce supramaximal contractions of the pelvic floor muscles, simulating thousands of Kegel exercises in a short session. These contractions strengthen the pelvic floor, improving bladder control and reducing prolapse symptoms without the need for surgery. EMSella therapy is performed on an outpatient basis, requires no recovery time, and eliminates surgical risks, making it an appealing option for patients with contraindications to surgery or those seeking conservative management [13,14,15].

Despite promising preliminary results, direct comparisons between EMSella therapy and surgical repair remain limited. Most studies focus on a single treatment modality, making it difficult for clinicians and patients to determine the most appropriate approach based on individual needs. While EMSella shows potential in improving UI and quality of life, its long-term efficacy in managing grade 2 cystocele compared to the anatomical correction achieved by anterior colporrhaphy remains unclear [16,17,18].

To address this gap, this study compares EMSella therapy and anterior colporrhaphy in managing UI associated with grade 2 cystocele. It evaluates objective clinical outcomes, such as prolapse reduction and urodynamic test normalization, alongside patient-reported measures like quality of life and satisfaction. The findings aim to guide clinical decision-making, providing evidence-based recommendations on the benefits and limitations of each approach to facilitate personalized treatment for affected patients.

## 2. Materials and Methods

### 2.1. Study Design and Patient Populations

**Figure 1:**
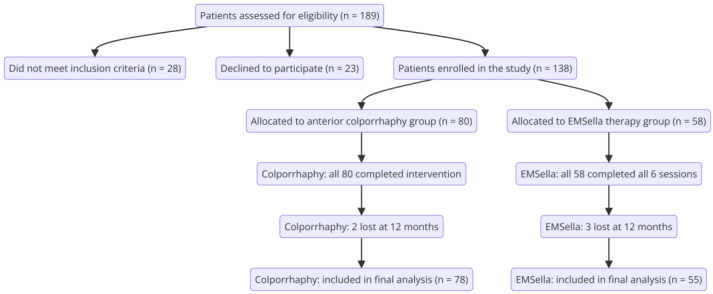
Participant flow diagram for clinical study enrollment and follow-up.

This prospective comparative study was conducted between January 2021 and December 2024 to evaluate the outcomes of two distinct treatment modalities for urinary incontinence associated with grade 2 cystocele: EMSella therapy and surgical anterior colporrhaphy. Pelvic organ prolapse was classified using the standardized Pelvic Organ Prolapse Quantification system, following IUGA/ICS recommendations. The study enrolled 133 female patients, with 78 treated surgically and 55 receiving EMSella therapy (Figure 1).

The surgical group was treated at two public healthcare institutions in Romania: Municipal Clinical Hospital “Dr. Alexandru Simionescu” Deva and County Emergency Clinical Hospital “Pius Brînzeu” Timișoara. In contrast, EMSella therapy was performed in three private clinics in Timișoara that specialize in non-invasive pelvic floor rehabilitation. The EMSella procedure was performed exclusively in a single private clinic, using the same chair model for all patients. The BTL EMSELLA^®^ device (BTL Industries Inc., Marlborough, MA, USA) was used for all sessions. This chair utilizes high-intensity focused electromagnetic (HIFEM) energy with a magnetic field strength of up to 2.5 Tesla and stimulation frequencies up to 30 Hz. Each 28-min session induces approximately 11,000 supramaximal pelvic floor muscle contractions. The device settings and protocols strictly followed the manufacturer’s clinical guidelines.

The surgical technique employed for anterior colporrhaphy followed a standardized approach aimed at anatomical restoration of the bladder’s support structures. The procedure began with an incision along the anterior vaginal wall, allowing access to the underlying fascia and bladder. The weakened endopelvic fascia was plicated with absorbable sutures to recreate adequate support for the bladder. Care was taken to ensure proper tensioning to avoid overcorrection while maintaining the natural vaginal anatomy [19].

Vaginal epithelium closure was performed with minimal tension, preserving vaginal length and ensuring postoperative comfort. The procedure was conducted by a multidisciplinary team comprising gynecologists and urologists with at least 10 years of experience in pelvic floor surgery. Both hospitals adhered to strict, standardized protocols to maintain consistency across all surgical cases.

EMSella therapy uses a high-intensity focused electromagnetic (HIFEM) stimulation device to induce supramaximal pelvic floor muscle contractions. Each patient underwent six 28-min sessions over three weeks, with sessions spaced evenly [6]. Patients were seated fully clothed on the EMSella chair, which delivered electromagnetic pulses targeting the pelvic floor [20].

The therapy was conducted under the supervision of certified physiotherapists, who ensured proper positioning and adherence to the manufacturer’s protocol. To isolate the effects of EMSella, no additional pelvic floor rehabilitation techniques, such as Kegel exercises or behavioral therapy, were permitted during the treatment period.

The primary outcomes included a reduction in prolapse severity, assessed using POP-Q staging [19], and bladder function improvement, evaluated through urodynamic tests. Secondary outcomes comprised prolapse recurrence, defined as a return to grade 2 or higher within 6 months or 1 year post-treatment, patient-reported quality of life measured by validated questionnaires such as PFDI-20 [20] and PFIQ-7 [21,22,23], and patient satisfaction, rated on a 5-point Likert scale [24].

### 2.2. Inclusion and Exclusion Criteria

Inclusion criteria:

- Women aged 40–75 years diagnosed with symptomatic stage II cystocele, confirmed by POP-Q examination, and presenting with stress urinary incontinence (SUI), as determined by standardized urodynamic assessments, including uroflowmetry, cough stress test, and urethral pressure profilometry. Diagnosis of SUI was confirmed by the presence of involuntary urine leakage during increased intra-abdominal pressure in the absence of detrusor overactivity.
- Women in menopause.
- Hemodynamic stability and controlled comorbidities.
- Written informed consent for participation in the study and completion of post-intervention questionnaires.
- The ICIQ-UI score confirmed the presence of urinary incontinence symptoms.
- Availability for post-intervention follow-up at 6 months and 1 year.
- Absence of prior surgical interventions on the pelvic floor.
- Patients treated according to standardized protocols for EMSella or anterior colporrhaphy.
- Accurate completion of clinical and follow-up records.
- A maximum of two vaginal deliveries.

Exclusion criteria:

- Patients with documented active infections (e.g., urinary tract or vaginal infections).
- Recent use (within the past 6 months) of antibiotics, probiotics, or other therapies influencing the microbiota.
- Pregnancy or breastfeeding.
- Undergoing hormonal therapy.
- Untreated endocrine disorders.
- Patients with documented hormonal imbalances.
- Patients with confirmed diagnoses of neurological disorders.
- History of spinal cord injuries affecting urinary function.
- Urinary incontinence associated with diabetic neuropathy.
- Patients with a BMI > 30 (obesity).
- Women engaging in high-impact sports.
- Active smokers or those with a long-term history of smoking.
- Women undergoing pharmacological treatments associated with urinary incontinence (e.g., diuretics, sedatives).
- Individuals with genetic conditions affecting urinary control.
- Women with documented diagnoses of connective tissue disorders with a genetic basis.
- Patients with documented anxiety or depression disorders.
- Women reporting significant stress as a trigger for symptoms.
- Urinary incontinence associated with psychosomatic disorders.
- Patients with urge urinary incontinence (UUI), mixed urinary incontinence (MUI), neurogenic bladder, or severe pelvic floor dysfunction requiring immediate surgical intervention.

### 2.3. Statistical Analysis

Statistical analyses were performed using GraphPad Prism 6 (GraphPad Software, San Diego, CA, USA). Descriptive statistics, including means, standard deviations, and percentages, were used to summarize the baseline characteristics of the study groups. Continuous variables, such as POP-Q scores, ICIQ-UI scores, and quality-of-life measures (PFDI-20 and PFIQ-7), were compared using paired *t*-tests, while categorical variables, including recurrence rates, patient satisfaction, and adverse events, were analyzed using chi-square tests. To assess changes over time for repeated measures at baseline, 6 months, and 1 year, we applied repeated measures ANOVA. Prior to analysis, we verified the assumptions required for parametric testing, including normality (using the Shapiro–Wilk test) and sphericity (assessed via Mauchly’s test). Statistical significance was considered at a two-tailed *p*-value of <0.05. Graphs and visual representations of trends were generated using GraphPad Prism 6.

### 2.4. Ethical Considerations

This study adheres to the highest ethical standards in medical research, in alignment with the Declaration of Helsinki. Ethical approval was obtained from the Institutional Review Boards (IRB) of all participating healthcare institutions, including Municipal Clinical Hospital “Dr. Alexandru Simionescu” Deva (201/14 December 2020) and County Emergency Clinical Hospital “Pius Brînzeu” Timișoara (107/21 December 2020). Informed consent was obtained from all participants before their enrollment in the study.

Participants were provided detailed information regarding the study objectives, methods, potential benefits, and associated risks. Consent forms were signed voluntarily, and participants retained the right to withdraw at any point without consequences to their medical care. Confidentiality and anonymity were maintained, with patient data anonymized before analysis to protect privacy.

Given the comparative nature of this study, all patients were treated under standardized and evidence-based protocols for both EMSella therapy and surgical anterior colporrhaphy. The medical team promptly addressed any adverse events or complications. Measures were taken to ensure equitable access to follow-up care and monitoring post-intervention, regardless of the treatment modality.

The study incorporated additional safeguards to minimize biases, including blinding data analysts to the treatment groups and ensuring an impartial selection process for the study population. Patients were informed about their treatment options based on clinical suitability and personal preferences, emphasizing shared decision-making in their care.

Lastly, innovative technologies, such as EMSella therapy, were carefully evaluated to ensure compliance with regulatory standards and manufacturer guidelines. Throughout the study, ongoing patient safety and satisfaction assessments were prioritized, reinforcing the commitment to ethical clinical practices.

## 3. Results

The study population’s baseline characteristics indicate a well-balanced distribution between the EMSella therapy and anterior colporrhaphy groups, facilitating a fair comparison of treatment outcomes (Table 1). Key demographic and clinical variables, such as age, body mass index (BMI), and symptom severity, showed no statistically significant differences, ensuring the cohorts’ comparability.

Pre-treatment scores for pelvic floor dysfunction (PFDI-20) and urinary incontinence severity (ICIQ-UI) were closely aligned, indicating that both groups had a similar burden of symptoms at baseline. Chronic constipation, a potential contributor to pelvic floor dysfunction, was also evenly distributed between the groups, further supporting the uniformity of clinical characteristics.

The duration of symptoms before treatment was comparable, reflecting a similar clinical history in both cohorts. This balanced distribution of demographic and clinical factors strengthens the study’s internal validity, allowing the focus to remain on the comparative evaluation of EMSella therapy versus anterior colporrhaphy for managing urinary incontinence.

Table 2 shows the analysis of objective parameters and highlights distinct differences in the clinical outcomes between the EMSella therapy and anterior colporrhaphy groups, providing valuable insights into the effectiveness of each intervention. Post-treatment outcomes revealed significant differences in prolapse reduction, with anterior colporrhaphy showing a superior improvement rate, as 88% of patients achieved stage 0 compared to 64% of EMSella patients improving to stage 1. Similarly, the proportion of patients without prolapse improvement was significantly higher in the EMSella group, emphasizing the enhanced efficacy of surgical intervention. Given that EMSella therapy primarily strengthens pelvic floor muscles without directly repositioning the bladder, achieving stage 0 was not a realistic outcome. Therefore, therapeutic success for EMSella was defined as a reduction to at least stage 1, whereas for anterior colporrhaphy, anatomical correction to stage 0 was expected. This distinction reflects the different mechanisms of action of the two treatments and has been clarified accordingly in the manuscript.

Bladder function, as assessed through urodynamic tests, also favored anterior colporrhaphy, with a greater normalization rate and fewer residual dysfunctions compared to EMSella therapy. Bladder function was assessed using urodynamic testing at baseline and post-treatment follow-ups at 6 months. The evaluations included uroflowmetry to measure voiding efficiency, post-void residual volume (PVR) assessment to detect incomplete bladder emptying, and cystometry to assess bladder compliance and detrusor overactivity. These tests provided objective measures of bladder function normalization and were critical in determining treatment efficacy and recurrence rates across both intervention groups. These findings underscore the surgical approach’s ability to address urinary dysfunction effectively.

Prolapse recurrence rates, evaluated at 6 months and 1 year, were lower in the anterior colporrhaphy group, particularly at the 1-year mark, where the difference reached statistical significance. These results highlight the long-term durability of surgical intervention over EMSella therapy in preventing prolapse recurrence.

Overall, the findings underscore the distinct advantages of anterior colporrhaphy in achieving superior anatomical and functional outcomes while reducing the risk of recurrence compared to EMSella therapy.

Table 3 compares the effectiveness of EMSella therapy and anterior colporrhaphy in treating prolapse and urinary incontinence in older women. The results indicate that anterior colporrhaphy is significantly more effective in reducing prolapse and improving bladder function. Surgically treated patients demonstrated better urethral closure pressure, lower post-void residual volume, and a greater reduction in detrusor overactivity. Regarding urinary incontinence, both treatments improved symptoms, but colporrhaphy had a stronger impact on bladder function. Post-treatment incontinence scores were significantly lower in the surgically treated group, with a higher percentage of patients achieving normalized bladder function. Significant *p*-values confirm the superiority of the surgical approach in most of the evaluated parameters.

The analysis of subjective parameters reveals significant differences in quality of life (QoL) and patient satisfaction outcomes between the EMSella therapy and anterior colporrhaphy groups (Table 4). While both groups presented comparable baseline scores for QoL metrics, including the PFDI-20 and PFIQ-7 scores, the improvements post-treatment were markedly more significant in the anterior colporrhaphy group.

Patients who underwent anterior colporrhaphy experienced more substantial reductions in both PFDI-20 and PFIQ-7 scores, indicating superior improvement in pelvic floor-related quality of life compared to EMSella therapy. The absolute changes in these scores were significantly more significant in the surgical group, reflecting its enhanced effectiveness in alleviating symptoms.

Patient satisfaction, measured using a Likert scale, was also higher among those treated with anterior colporrhaphy. While both groups reported high satisfaction levels, the proportion of patients who were “very satisfied” was slightly higher in the surgical group, although this difference did not reach statistical significance. Notably, a more significant percentage of anterior colporrhaphy patients preferred the same treatment in the future, suggesting higher confidence in the surgical approach.

Overall, the findings highlight that anterior colporrhaphy delivers more pronounced improvements in quality of life and fosters greater patient satisfaction and treatment preference compared to EMSella therapy. These results underscore the importance of tailored approaches addressing clinical and subjective aspects of urinary incontinence management.

The analysis of complications and adverse effects highlights significant differences between EMSella therapy and anterior colporrhaphy regarding overall safety and tolerability. The overall rate of adverse effects was notably higher in the anterior colporrhaphy group, reflecting the more invasive nature of the surgical intervention (Table 5).

Temporary irritation was more frequently reported in the EMSella group, likely due to localized tissue stimulation associated with the non-invasive nature of the therapy. In contrast, anterior colporrhaphy patients experienced a higher incidence of infections, such as urinary tract or vaginal infections, emphasizing the need for vigilant postoperative monitoring in surgical cases. Dyspareunia was reported exclusively in the anterior colporrhaphy group, reflecting its impact on sexual function, a common concern following pelvic reconstructive surgery. Patient-reported outcomes and adverse effects were assessed 7 days after the intervention.

These findings underscore the trade-offs between non-invasive and surgical interventions in managing urinary incontinence and prolapse. While EMSella therapy offers a favorable safety profile with fewer complications, anterior colporrhaphy demonstrates a higher complication rate that must be weighed against its potentially superior efficacy in advanced cases. These results emphasize the importance of individualized treatment selection to balance effectiveness and safety for each patient.

The analysis of recovery parameters and their impact on patients’ social and professional lives reveals stark differences between EMSella therapy and anterior colporrhaphy. EMSella therapy demonstrated a markedly shorter recovery period, with most patients resuming daily activities within a week, compared to a significantly longer recovery time in the surgical group. This distinction underscores the non-invasive nature of EMSella therapy, which minimizes downtime and facilitates a quicker return to routine life (Table 6).

The mean recovery time was substantially shorter for EMSella therapy, highlighting its suitability for patients seeking minimal disruption to their daily lives. In contrast, anterior colporrhaphy required extended recovery periods, with a significant proportion of patients resuming daily activities only after two weeks or more, reflecting the invasive nature and postoperative requirements of the surgical approach.

Social and professional disruptions were also more pronounced in the anterior colporrhaphy group. Patients undergoing surgery reported higher rates of temporary interruptions in social engagements and professional activities, with a significant majority temporarily unable to work. In contrast, EMSella therapy resulted in minimal disruptions, aligning with its less invasive profile and faster recovery.

Patient-reported comfort levels, assessed on a Likert scale, were higher among EMSella therapy participants, reflecting its better overall tolerability and convenience. These findings collectively emphasize the advantages of EMSella therapy in minimizing recovery time and preserving patients’ social and professional functionality. At the same time, anterior colporrhaphy, despite its efficacy, necessitates more extensive recovery and adaptation. Such insights are critical for tailoring treatment strategies to individual patient priorities and lifestyles.

## 4. Discussion

This study evaluates the effectiveness of EMSella therapy compared to anterior colporrhaphy in managing grade 2 cystocele associated with stress urinary incontinence (SUI). Contributing to the medical literature, the research highlights the differences between invasive and non-invasive approaches, providing relevant data for personalized clinical decision-making. The analysis covers pelvic function outcomes, quality of life, treatment safety, and post-intervention recovery, emphasizing the advantages and limitations of each method.

One of the key findings is the significant difference in prolapse reduction between the two treatment methods. We found that 88.46% of patients treated with anterior colporrhaphy achieved complete anatomical correction (POP-Q stage 0), compared to only 63.63% of those treated with EMSella, who reached only stage 1. These results confirm the superiority of surgical intervention in correcting moderate-to-severe prolapse [25], offering more effective anatomical restoration than non-invasive therapies [26].

Several studies support that surgical prolapse repair using native tissue has high long-term success rates, significantly improving pelvic support and urinary continence [27,28,29]. Reports indicate success rates between 70 and 90% for anterior colporrhaphy, confirming its effectiveness in preventing prolapse progression and alleviating symptoms [30,31]. Additionally, the literature shows that 85% of patients reported a significant improvement in symptoms two years postoperatively, highlighting the durability of surgical benefits [32,33]. However, prolapse recurrence remains a concern, especially for patients with risk factors such as advanced age, high body mass index, or severe weakening of pelvic support structures [34,35]. Recent studies report recurrence rates of 15–30% within the first five years postoperatively, emphasizing the need for long-term follow-up and preventive measures [36,37].

By comparison, EMSella showed lower efficacy in anatomical correction but provided significant functional improvements. The treatment strengthens pelvic floor muscles by using high-intensity focused electromagnetic (HIFEM) stimulation, improving urinary control [16]. Approximately 60–70% of patients reported improved urinary continence, but its effectiveness decreases in advanced prolapse cases [38]. EMSella’s benefits may diminish over time without maintenance sessions, with some patients experiencing symptom recurrence after one year. This suggests that EMSella should be combined with other conservative methods, such as pelvic floor muscle exercises or vaginal pessaries [17,39].

Our findings highlight the superior efficacy of anterior colporrhaphy compared to EMSella therapy in improving both prolapse severity and urinary function. The significant differences in post-treatment POP-Q staging and urodynamic parameters confirm that surgical intervention provides better structural and functional outcomes. These results align with previous studies demonstrating that anterior colporrhaphy leads to greater anatomical correction and long-term symptom relief in patients with pelvic organ prolapse and urinary incontinence [26]. Additionally, our findings support the existing literature indicating that non-surgical approaches, such as EMSella, may offer symptomatic relief but are less effective in restoring bladder compliance and urethral function [40]. The significant reduction in ICIQ-UI scores in both groups suggests that while conservative therapies can improve quality of life, surgical correction remains the gold standard for sustained functional recovery. Future studies should further explore the long-term durability of these interventions, particularly in high-risk populations.

In our study, anterior colporrhaphy had a greater impact on quality of life compared to EMSella. The significant reduction in PFDI-20 and PFIQ-7 scores in the surgical group indicates substantial symptom relief for pelvic organ prolapse and urinary incontinence. The literature supports these findings, confirming that surgical interventions remain the most effective option for restoring pelvic function and improving quality of life [23].

A large study demonstrated that surgical pelvic organ prolapse procedures significantly reduce symptoms and provide long-term benefits, with high patient satisfaction rates. Furthermore, 85% of surgically treated women reported a significant quality-of-life improvement even five years postoperatively [41]. These findings confirm that surgery surpasses non-invasive symptom reduction and pelvic function restoration therapies. However, anterior colporrhaphy involves surgical risks and a longer recovery period. Patients treated with EMSella reported significant quality-of-life improvements, mainly due to the absence of postoperative pain and the short recovery time. Unlike surgery, which requires healing and postoperative restrictions, EMSella allows for immediate resumption of daily activities, contributing to higher patient compliance and satisfaction.

A study showed that EMSella significantly improves urinary continence and patient satisfaction, particularly in those who prefer a minimally invasive approach or have contraindications to surgery [16]. However, the lack of long-term anatomical correction and the need for maintenance sessions limit its effectiveness compared to surgical methods.

In terms of safety, EMSella had a lower rate of adverse effects than anterior colporrhaphy. In the surgical group, 14.10% of patients developed dyspareunia and urinary or vaginal infections postoperatively. These complications are frequently reported in the medical literature, with dyspareunia rates between 10 and 20% after colporrhaphy due to vaginal scarring and pelvic structural changes [42,43].

Conversely, EMSella was associated with minor and transient adverse effects (14.54%), such as temporary irritation, muscle discomfort, or involuntary contractions during treatment. These effects are linked to EMSella’s mechanism of action, which induces involuntary muscle contractions without affecting surrounding tissues.

EMSella therapy has a high safety profile, requiring no anesthesia, incisions, or recovery time, making it an attractive option for patients who wish to avoid invasive procedures. A study has shown that EMSella allows for a quicker return to daily activities, with most patients resuming their usual routine within a week, in contrast to the longer recovery period required after colporrhaphy [16,44]. Thus, this therapy represents a viable alternative for patients who prioritize post-treatment comfort and rapid recovery. However, anterior colporrhaphy remains the gold standard for definitive anatomical correction of prolapse, and treatment selection should be individualized, taking into account the patient’s clinical status, expectations, and preferences. This study benefits from a robust design, providing a comprehensive comparative analysis of both clinical and subjective outcomes for EMSella therapy and anterior colporrhaphy in managing urinary incontinence associated with grade 2 cystocele. The use of validated assessment tools, such as the POP-Q system, urodynamic tests, and PFDI-20 and PFIQ-7 scores, ensures the reliability of outcome measurements. The inclusion of recovery time and the impact on social and professional life adds a practical dimension, making the findings relevant to patient-centered care. Furthermore, the study was conducted across two clinical centers, enhancing the generalizability of its results and reducing the potential for center-specific biases. The standardized treatment protocols and adherence to ethical research practices further strengthen the study’s credibility. By examining both invasive and non-invasive treatment options, the study addresses a critical gap in the literature and provides valuable insights for personalized clinical decision-making.

Despite its strengths, the study is limited by its two-center design, which, although an improvement over single-center studies, may still restrict the external validity of its findings to other healthcare settings with varying protocols and patient demographics. The relatively short follow-up period of one year limits the ability to draw conclusions about the long-term efficacy and durability of the interventions. The retrospective nature of the analysis introduces potential biases and confounding variables, as data collection was not prospectively planned. Additionally, the exclusion of patients with prior treatments or significant comorbidities reduces the representativeness of the study population, potentially limiting its applicability to broader clinical scenarios. The absence of advanced imaging techniques or microbiome analyses also restricts the study’s ability to explore deeper pathophysiological mechanisms underlying the observed outcomes, which could have provided more nuanced insights into treatment efficacy. A potential bias may arise from the fact that treatment allocation was influenced by the type of institution (public hospital vs. private clinic), possibly reflecting differences in patient lifestyle or physical condition. Although a post hoc power analysis indicated a moderate statistical power (≈51%) for some outcomes, the study provides valuable comparative clinical data on invasive and non-invasive approaches, supporting its relevance in guiding personalized treatment decisions.

## 5. Conclusions

Anterior colporrhaphy showed superior anatomical correction and bladder function improvement, with more patients achieving complete prolapse resolution. EMSella therapy, while less effective anatomically, remains a safe and non-invasive alternative for patients with mild-to-moderate prolapse or surgical contraindications. Both treatments improved quality of life, but surgery led to greater reductions in PFDI-20 and PFIQ-7 scores. EMSella had fewer complications, no dyspareunia, and faster recovery, though it showed higher recurrence rates at one year. These findings highlight the need for individualized treatment based on patient profile and suggest further research into long-term outcomes and combined non-invasive strategies.

## Figures and Tables

**Table 1:** Baseline characteristics of patients undergoing EMSella therapy and anterior colporrhaphy.

Characteristics	EMSella Therapy (n = 55)	Anterior Colporrhaphy (n = 78)	*p*-Value
Mean age (years)	49.5 ± 6.9	51.2 ± 7.0	0.167
Body mass index (BMI)	25.1 ± 3.8	24.4 ± 4.0	0.312
PFDI-20 score (pre-treatment)	79.2 ± 13.1	80.5 ± 12.9	0.570
Chronic constipation (%)	12 (21.81%)	16 (20.51%)	0.761
Severity of incontinence (ICIQ-UI, mean score)	13.4 ± 3.3	13.7 ± 3.4	0.612
Symptom duration (years)	3.8 ± 1.9	4.0 ± 2.0	0.563

**Table 2:** Objective parameters in EMSella therapy vs. anterior colporrhaphy for Grade 2 cystocele.

Objective Parameter	EMSella Therapy (n = 55)	Anterior Colporrhaphy (n = 78)	*p*-Value
1. Prolapse Reduction (POP-Q)			
Post-treatment improvement	To stage 1 or better: 35 (63.63%)	To stage 0: 69 (88.46%)	<0.001 *
No improvement	20 (36.36%)	9 (11.53%)	<0.001 *
2. Bladder Function (Urodynamic Tests)			
Post-treatment normalization	55% (30/55)	56 (71.78%)	0.044 *
Residual dysfunction post-treatment	45% (25/55)	22 (28.20%)	0.041 *
3. Prolapse Recurrence			
Recurrence at 6 months	10 (18.18%)	7 (8.97%)	0.094
Recurrence at 1 year	17 (30.90%)	11 (14.10)	0.032 *

* Statistically significant *p*-value.

**Table 3:** Objective parameters for prolapse and urinary incontinence outcomes.

Parameter	MSella Therapy (n = 55)	Anterior Colporrhaphy (n = 78)	*p*-Value
Prolapse Reduction (POP-Q Staging)			
Post-treatment stage 0 (no prolapse)	18 (32.72%)	66 (84.61%)	<0.001 *
Post-treatment stage 1	24 (43.63%)	10 (12.82%)	0.002 *
No improvement (stage ≥ 2)	13 (23.63%)	2 (2.56%)	<0.001 *
Urodynamic Test Results			
Maximum urethral closure pressure (cmH_2_O)	38.5 ± 7.2	42.8 ± 6.5	0.021 *
Post-void residual volume (mL)	62.4 ± 14.3	8.1 ± 11.8	0.017 *
Bladder compliance (mL/cmH_2_O)	2.7 ± 5.1	7.3 ± 4.8	0.039 *
Detrusor overactivity (%)	16 (29.09%)	12 (15.38%)	0.044 *
Urinary incontinence severity			
Q-UI Score (pre-treatment)	13.4 ± 3.3	13.7 ± 3.4	0.612
ICIQ-UI Score (post-treatment)	8.2 ± 2.5	5.1 ± 2.1	<0.001 *
Normalized Bladder Function (%)	30 (54.54%)	56 (71.79%)	0.044 *

* *p* Value < 0.05.

**Table 4:** Subjective parameters in EMSella therapy vs. anterior colporrhaphy for Grade 2 cystocele.

Subjective Parameter	EMSella Therapy (n = 55)	Anterior Colporrhaphy (n = 78)	*p*-Value
1. Quality of Life (QoL)			
Baseline PFDI-20 score	79.2 ± 13.1	80.5 ± 12.9	0.570
Post-treatment PFDI-20 score	50.7 ± 10.5	40.4 ± 11.2	<0.001 *
Change in PFDI-20 score (absolute)	28.5 ± 10.2	40.1 ± 12.5	<0.001 *
Baseline PFIQ-7 score	75.8 ± 14.3	77.2 ± 13.7	0.569
Post-treatment PFIQ-7 score	52.1 ± 11.0	41.3 ± 10.7	<0.001 *
Change in PFIQ-7 score (absolute)	23.7 ± 9.8	35.9 ± 11.5	<0.001 *
2. Patient Satisfaction			
Overall satisfaction (Likert scale 1–5)	4.1 ± 0.7	4.5 ± 0.5	0.002 *
Patients “very satisfied” (score 4–5)	45 (81.81%)	71 (91.02%)	0.091
Preference for the same treatment (%)	41 (74.54%)	69 (88.46%)	0.043 *

* Statistically significant *p*-value.

**Table 5:** Complications and adverse effects in EMSella therapy vs. anterior colporrhaphy.

Complication/Adverse Effect	EMSella Therapy (n = 55)	Anterior Colporrhaphy (n = 78)	*p*-Value
1. Overall rate of adverse effects (%)	8 (14.54%)	27 (34.61%)	0.011 **
2. Specific Complications			
Temporary irritation (%) *	5 (9.09%)	4 (5.12%)	0.341
Infections (e.g., UTI, vaginal) (%)	2 (3.63%)	11 (14.10%)	0.025 **
Dyspareunia (%)	0 (0%)	11 (14.10%)	<0.001 **

* Temporary irritation in the EMSella group refers to transient skin discomfort, mild muscular soreness, or localized hypersensitivity due to electromagnetic stimulation; in the anterior colporrhaphy group, it refers to post-surgical tissue inflammation, local edema, or sensitivity associated with wound healing; ** statistically significant *p*-value.

**Table 6:** Recovery duration and patient comfort in EMSella therapy vs. anterior colporrhaphy.

Parameter	EMSella Therapy (n = 55)	Anterior Colporrhaphy (n = 78)	*p*-Value
1. Time to Resume Daily Activities			
<1 week (%)	50 (90.90%)	9 (11.53%)	<0.001
1–2 weeks (%)	5 (9.09%)	18 (23.09%)	<0.001
>2 weeks (%)	0 (0%)	51 (65.38%)	<0.001
Mean recovery time (days)	2.3 ± 1.1	18.7 ± 6.5	<0.001
2. Impact on Social and Professional Life			
Temporary disruption of social activities (%)	8 (14.54%)	30 (38.46%)	0.002
Temporary inability to work (%)	4 (7.27%)	63 (80.76%)	<0.001
Patient-reported comfort (Likert scale 1–5)	4.6 ± 0.4	3.8 ± 0.7	<0.001

## Data Availability

The data are available from the corresponding author of the study. You may contact the corresponding author for further details and access to the relevant data. Additionally, a copy of the data is also stored in our hospital’s records. If you require access to the data from our hospital, please feel free to reach out to our data management department.
